# Acid Solution Is a Suitable Medium for Introducing QX-314 into Nociceptors through TRPV1 Channels to Produce Sensory-Specific Analgesic Effects

**DOI:** 10.1371/journal.pone.0029395

**Published:** 2011-12-28

**Authors:** He Liu, Hong-Xing Zhang, Hui-Yan Hou, Xian-Fu Lu, Jing-Qiu Wei, Chun-Guang Wang, Li-Cai Zhang, Yin-Ming Zeng, Yong-Ping Wu, Jun-Li Cao

**Affiliations:** 1 Jiangsu Province Key Laboratory of Anesthesiology, Xuzhou Medical College, Xuzhou, China; 2 Jiangsu Province Key Laboratory of Anesthesia and Analgesia Application Technology, Xu Zhou, China; 3 Department of Anesthesiology, Affiliated Hospital of Xuzhou Medical College, Xu Zhou, China; Hokkaido University, Japan

## Abstract

**Background:**

Previous studies have demonstrated that QX-314, an intracellular sodium channel blocker, can enter into nociceptors through capsaicin-activated TRPV1 or permeation of the membrane by chemical enhancers to produce a sensory-selective blockade. However, the obvious side effects of these combinations limit the application of QX-314. A new strategy for targeting delivery of QX-314 into nociceptors needs further investigation. The aim of this study is to test whether acidic QX-314, when dissolves in acidic solution directly, can enter into nociceptors through acid-activated TRPV1 and block sodium channels from the intracellular side to produce a sensory-specific analgesic effect.

**Methodology/Principal Findings:**

Acidic solution or noradrenaline was injected intraplantarly to induce acute pain behavior in mice. A chronic constrictive injury model was performed to induce chronic neuropathic pain. A sciatic nerve blockade model was used to evaluate the sensory-specific analgesic effects of acidic QX-314. Thermal and mechanical hyperalgesia were measured by using radiant heat and electronic von Frey filaments test. Spinal Fos protein expression was determined by immunohistochemistry. The expression of p-ERK was detected by western blot assay. Whole cell clamp recording was performed to measure action potentials and total sodium current in rats DRG neurons. We found that pH 5.0 PBS solution induced behavioral hyperalgesia accompanied with the increased expression of spinal Fos protein and p-ERK. Pretreatment with pH 5.0 QX-314, and not pH 7.4 QX-314, alleviated pain behavior, inhibited the increased spinal Fos protein and p-ERK expression induced by pH 5.0 PBS or norepinephrine, blocked sodium currents and abolished the production of action potentials evoked by current injection. The above effects were prevented by TRPV1 channel inhibitor SB366791, but not by ASIC channel inhibitor amiloride. Furthermore, acidic QX-314 employed adjacent to the sciatic nerve selectively blocked the sensory but not the motor functions in naïve and CCI mice.

**Conclusions/Significance:**

Acid solution is a suitable medium for introducing QX-314 into nociceptors through TRPV1 channels to produce a sensory-specific analgesic effect.

## Introduction

Peripheral nerves serve mainly three functions, including sensation, movement and autonomic modulation. Most local anesthetics (LA) currently employed in clinics produce a blockade of sensory, motor and autonomic nerves via blocking voltage-gated Na^+^ channels to induce analgesia, muscle relaxation (immobility) and loss of peripheral autonomic modulation [1], [2]. However, in some clinical cases, LA that selectively block of sensory nerves are more ideal.

QX-314, a membrane-impermeable quaternary lidocaine derivative, has no effect on neuronal sodium channels with extracellular application but does block sodium channels when applied intracellularly. Woolf and colleagues reported that a long-lasting sensory-selective blockage was produced by co-administration of QX-314 and capsaicin, a transient receptor potential cation channel, subfamily V, member 1 (TRPV1) agonist [3], [4], [5], [6], [7]. TRPV1 channels are only expressed on the nociceptors. Activating TRPV1 channels by capsaicin allowed QX-314 to enter into TRPV1 positive neurons only, where it then blocks the sodium channels from the intracellular side and then produces an analgesic effect without interfering with motor function [3], [4], [5], [6], [7]. Recent findings have indicated that co-application of chemical membrane permeability enhancers Tween 20 or octyltrimethylammonium bromide and QX-314 also produced a similar effect [7]. However, application of capsaicin or chemical permeability enhancers would produce some adverse effects including acute pain and potential neurotoxicity et al [8], [9], [10]. These combinations are also inconvenient for clinical use. Therefore, investigation of a new strategy for targeting delivery of QX-314 into nociceptors is needed.

The TRPV1 channels are non-selective cation channels that serve as a pain-sensing transducer and express peripherally in primary afferent nociceptors, which can be activated by capsaicin, noxious heat (>43°C), protons (pH<5.9) and various inflammatory mediators [11], [12], [13], [14]. Most LA applied widely in clinical settings now is dissolved in an acidic solution (pH 3.3∼5.5). So, we want to know whether acidic QX-314 (directly dissolved in pH 5.0 PBS) could be used to selectively target nociceptors and produce sensory-selective blockage via proton activated-TRPV1 channels, as capsaicin did.

## Materials and Methods

### Animals

Adult male Kunming mice (18–22 -g) and Sprague-Dawley rats (6–8 weeks) employed in present studies were provided by Experimental Animal Center of Xuzhou Medical College. Mice were housed with controlled relative humidity (20–30%) and temperature (23±2 -°C), under a 12 h light-dark cycle (light on 08:00 to 20:00), and with free access to food and water *ad libitum*. Before experiments, the animals were allowed to habituate to the housing facilities for 7 days and efforts were made to limit distress to the animals. All experimental protocols were approved by the Animal Care and Use Committee of Xuzhou Medical College (Xuzhou, Jiangsu Province, China) and according to the Declaration of National Institutes of Health *Guide for Care and Use of Laboratory Animals* (Publication No. 80–23, revised 1996).

### Drug application

N-(2, 6-dimethylphenyl carbamoylmethyl) triethylammonium chloride (QX-314) and 5-(N-Methyl-N-isobutyl) amiloride, a non–selective acid-sensing ion channel (ASIC) antagonist, were purchased from Sigma-Aldrich (St. Louis, MO). N-(3-Methoxyphenyl)-4-chlorocinnamide (SB366791), a potent and selective TRPV1 antagonist, was purchased from Enzo Life Sciences (San Diego, CA). SB366791 was dissolved in dimethyl sulfoxide (DMSO) for stock solution (25-mg/ml) and other drugs in PBS. The final DMSO concentration was less than 1% for behavior test and 0.1% for electrophysiological experiments. PBS was titrated with NaOH or HCl as needed. All doses of drugs were based on the results of preliminary experiments. The doses of each drug and time points of treatment are presented in parts of the results and figure legends.

Mice were gently restrained, and all drugs or vehicles were administered in a volume of 10-µl into the plantar surface of the right hind paw using a 25-µl Hamilton syringe with a 28-gauge needle. The needle was inserted into the plantar skin proximal to the midpoint of the hind paw and advanced about 10-mm so that it reached the midpoint of the hind paw, then the solution was injected to form a bleb which disappeared within 10-min.

### Measurement of thermal hyperalgesia

Thermal hyperalgesia was measured by the IITC Plantar Analgesia Meter (IITC Life Science Inc., Victory Blvd Woodland Hills, CA) for paw withdrawal latency according to the method described by Hargreaves et al [15]. In brief, mice were placed in transparent acrylic enclosures (7×9×11-cm) with a glass plate, and allowed to acclimatize to their environment for 1-h before testing in a temperature-controlled and noise-free room (23±2-°C). The high-intensity, movable radiant heat source was placed underneath the glass and focused onto the plantar surface of each hind paw. The nociceptive endpoint in the radiant heat test was characteristic lifting or licking of the hind paw. The time from onset of radiant heat to endpoint was considered as the paw withdrawal latency (PWL). The radiant heat intensity was adjusted at the beginning of the experiment to obtain basal PWL of 12∼15-s, and kept constant thereafter. An automatic 25-s cutoff was used to prevent tissue damage. Each animal was tested 3 times on each hind paw at intervals of 5-min.

### Measurement of mechanical allodynia

Mechanical allodynia was assessed by using electronic von Frey filaments (IITC Life Science Inc., Victory Blvd Woodland Hills, CA). Animals were placed in individual plastic boxes (20×25×15-cm) on a metal mesh floor and allowed to acclimate for 1-h. The filaments were presented, in ascending order of strength, perpendicular to the plantar surface with sufficient force to cause slight bending against the paw and held for 6–8-s. Brisk withdrawal or paw flinching was considered as positive responses. The paw withdrawal threshold (PWT) was determined by sequentially increasing and decreasing the stimulus strength (the “up- and -down” method), and the data were analyzed using the nonparametric method of Dixon, as described by Chaplan et al [16].

### Chronic constrictive injury (CCI) model

CCI model was performed following the method of Bennett and Xie [17]. In brief, mice were anesthetized with sodium pentobarbital (40-mg/kg, intraperitoneal injection). The left sciatic nerve was exposed at mid-thigh level through a small incision and a unilateral constriction injury just proximal to the trifurcation was performed with three loose ligatures using a 5-0 silk thread (spaced at a 1-mm interval). In sham-operated animals, the nerve was exposed but not ligated. The incision was closed in layers, and the wound was treated with antibiotics.

### Sciatic nerve blockade model

According to the method reported by Leszczynska and Kau [18], all mice were placed in the middle of a 20×25-cm inverted mesh and acclimatized to climb to the outside and over the edge of the mesh, and mice could climb on mesh with all four limbs before experiments. Mice were slightly restrained and drugs were injected into the area of the popliteal fossa of the left hind limb using a 50-µl Hamilton syringe with a 28-gauge needle. After injection, mice were placed onto the mesh, and primary endpoint was the time to loss of ability to hang on to the inverted mesh with the injected hind limb, which was tested at 5, 10, 15, 20, 25 and 30 -min after injection.

### Immunohistochemistry

Mice were anesthetized with sodium pentobarbital (60-mg/kg, intraperitoneal injection) and subjected to sternotomy followed intracardial perfuse with 20-ml saline followed by 100-ml 4% ice-cold paraformaldehyde in 0.1-mol/L phosphate buffer. The spinal cord of L_4–5_ was removed, post-fixed in 4% paraformaldehyde for 3-h, and subsequently allowed to equilibrate in 30% sucrose in phosphate buffer overnight at 4-°C. Thirty-µm transverse series sections were cut on a cryostat and stored in phosphate buffer. After washing in phosphate buffer saline, the tissue sections were incubated in phosphate buffer saline containing 5% normal goat serum and 0.3% TritonX-100 at room temperature for 30-min. For the Fos protein assay, the sections were incubated in primary polyclonal rabbit-anti-Fos antibody (1∶1000) (Santa Cruz Biotechnology, Santa Cruz, CA) at 4-°C for 48-h. The sections were then incubated in biotinylated goat anti-rabbit (1∶200) at 37-°C for 1-h and in avidin-biotin-peroxidase complex (1∶100) (Vector Labs, Burlingame, CA) at 37-°C for 2-h. Finally, the sections were treated with 0.05% diaminobenzidine for 5–10-min. Sections were rinsed in phosphate buffer saline to stop the reaction, mounted on gelatin-coated slides, air-dried, dehydrated with 70%–100% alcohol, cleared with xylene, and cover-slipped for microscopic examination. To analyze the change of Fos protein expression, we examined 5 L_4–5_ spinal cord sections per animal, selecting the sections with the greatest number of positive neurons. For each animal, we recorded the total number of positive neurons in the bilateral spinal cord I∼V lamina. All positive neurons were counted without considering the intensity of the staining.

### Western blot analysis

The spinal cords of the mice were quickly extracted and stored in liquid nitrogen. Tissue samples were homogenized in lysis buffer containing (in mM, pH 7.4): Tris 20.0, sucrose 250.0, Na_3_VO_4_ 0.03, MgCl_2_ 2.0, EDTA 2.0, EGTA 2.0, phenylmethylsulfonyl fluoride 2.0, dithiothreitol 1.0, and protease inhibitor cocktail 0.02% (v/v). The homogenates were centrifuged at 5000-g for 30-min at 4-°C. The supernatant was collected and protein concentration was performed according to the Bradford (1976) method using bovine serum albumin as a standard [19]. The protein samples were stored at −80-°C.

Protein samples were dissolved in 4 ×sample buffer (in mM, pH 6.8): Tris-HCl 250.0, Sucrose 200.0, Dithiothreitol 300.0, 0.01% Coomassie brilliant blue-G, and 8% sodium dodecyl sulfate, and denatured at 95-°C for 5-min. Then the equivalent amounts of protein (80-µg) were separated using 10% sodium dodecyl sulfate-polyacrylamide gel electrophoresis and transferred onto nitrocellulose membrane. In addition, the gels stained with Coomassie blue were used to confirm the equal amounts of protein loaded on each lane. The membranes were incubated overnight at 4-°C with the primary polyclonal rabbit anti-p-ERK1/2 or anti-ERK1/2 antibody (1∶700, Bioword, St. Louis Park, MN). The specificity for p-ERK antibodies was confirmed by loss of bands in the absence of primary antibodies. The membranes were extensively applied with Tris-Buffered Saline Tween-20 and incubated for 2-h with the secondary antibody conjugated with alkaline phosphatase (1∶500, Santa Cruz, CA) at room temperature. The immune complexes were detected by using a nitro blue tetrazolium/5-bromo-4-chloro-3-indolyl phosphate assay kit (Sigma, St. Louis, MO). Western blot densitometry analysis of signal intensity was performed using Adobe Photoshop software (Adobe, San Jose, CA) and phosphorylation levels of ERK from densitometry were normalized to total ERK. The blot density from control groups was set as 100%.

### Electrophysiological recordings

Electrophysiological recordings from DRG neurons were performed with whole cell current- clamp recording methods similar to previous studies [3]. Briefly, DRGs of 6-8-week-old Sprague-Dawley rats were removed and placed in DMEM containing 1% penicillin-streptomycin (Sigma, St. Louis, MO), treated for 90-min with 5-mg/ml collagenase, 1-mg/ml Dispase II (Roche), then with 0.25% trypsin for 7-min, followed by 2.5% trypsin inhibitor. Cells were triturated in the presence of DNAase I inhibitor (50-U), centrifuged through 15% BSA (Sigma, St. Louis, MO), resuspended in 1-ml of Neurobasal medium (Sigma, St. Louis, MO), 10-µm AraC, 50-ng/ml nerve growth factor and 2-ng/ml glial cell line-derived neurotrophic factor and plated onto 35-mm tissue culture dishes (Becton Dickinson) coated with 500-µg/ml polylysine and 5-mg/ml laminin, at 8,000–9,000 cells per dish. Cultures were incubated at 37-°C under 5% CO_2_. Recordings were made at room temperature within 48-h of plating. The artificial cerebrospinal fluid used as bath solution was composed of 128 NaCl, 3 KCl, 1.25 NaH_2_PO_4_, 10 D-glucose, 24 NaHCO_3_, 2 CaCl_2_, and 2 MgSO_4_, oxygenated with 95% O_2_ and 5% CO_2_ (in mM, pH 7.35, 295–300 -mOsm).

Whole cell patch-clamp recordings were made with an Axoclamp 700B amplifier (Molecular Devices) from small DRG neurons. Glass electrodes (4–6-MΩ) were fabricated with a Flaming/Brown micropipette puller (P-97, Sutter instruments) and were filled with an internal solution containing (in mM: 120 potassium gluconate, 20 KCl, 1 CaCl_2_, 2 MgCl_2_, 10 EGTA, 2 Mg-ATP, 0.5 Na_2_-GTP, and 10 HEPES (pH 7.2, 280–290 -mOsm). Action potentials were evoked by 25-ms depolarizing current pulses with 100-pA step amplitude in current patch clamp mode, and total sodium current was recorded by applying a depolarizing voltage pulse from the holding potential of −65 mV to −5 mV in the presence of potassium and calcium channel blockers in the voltage-clamp mode in DRG neurons. Data were low-pass filtered at 2-kHz, digitized at a sampling rate of 10-kHz. Drugs were applied through a solution perfusion system.

### Statistical analysis

Data are expressed as mean ± S.E.M. Statistical analysis of more than two groups was performed using one-way analysis of variance (ANOVA) followed by a Tukey's post hoc test. The significance of any differences in thermal latency threshold in the behavior testing was assessed using two-way ANOVA. Time was treated as a ‘within subjects’ factor and ‘treatment’ was treated as a ‘between subjects’ factor. The area under the pain threshold change versus time curve was calculated by GraphPAD Prism5 (Graph Pad Software Inc., San Diego, CA) in some behavioral testss. Statistical analyses of data were generated using GraphPAD Prism5. All p values given are based on two-tailed tests. *P*<0.05 was considered as statistically significant.

## Results

### Acidic PBS induces TRPV1-mediated hyperalgesia and spinal neuron sensitization

**Figure 1:**
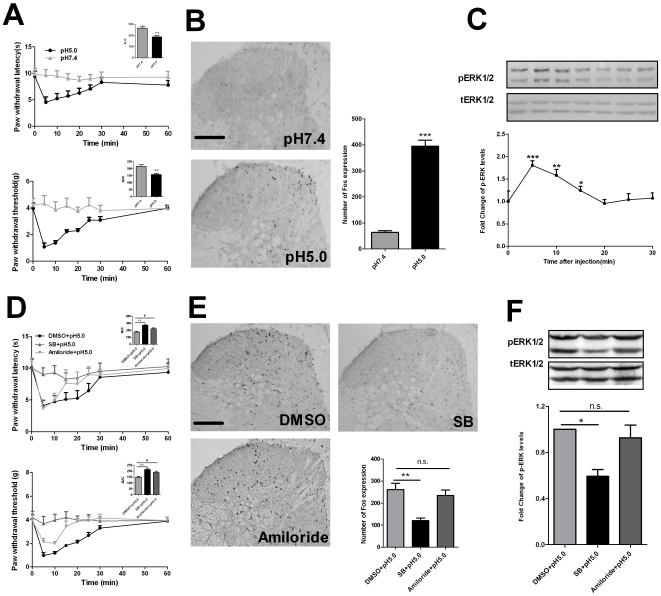
Intraplantar acid injection produced TRPV1-mediated time-dependent behavioral hyperalgesia and spinal neuron sensitization. (A) Intraplantar injection of pH 5.0 PBS (10-µl), not pH 7.4 PBS (10-µl), produced thermal (top) and mechanical (bottom) hyperalgesia. *P<0.05, from 5 to 25-min time point, pH 5.0 PBS group vs. pH 7.4 PBS group. Inset figures showed that the calculated area under curve (AUC) (0∼60-min) in paw-withdrawal latency (PWL) test was significantly decreased in pH 5.0 PBS group. **P<0.01 compared with pH 7.4 PBS group, n = 8 mice in each group. (B) Representative immunohistochemical staining and quantitative data of Fos in the spinal cord of mice. Intraplantar injection of pH 5.0 PBS (10-µl), but not pH 7.4 PBS (10-µl), increased spinal Fos protein expression. Quantitative data indicats the number of Fos positive neurons in the spinal cord in each group. ***P<0.001 compared with pH 7.4 PBS group, n = 6 mice in each group. Scale bar = 100-µm. (C) The representative bands (top) for the expression of p-ERK at different time points after injection of pH 5.0 PBS (10-µl) and the quantitative data (bottom) for the expression of p-ERK. The fold change for the density of p-ERK is normalized to total-ERK for each sample. The fold change for the density of p-ERK levels in the 0-time point group was set at 1 for quantification. Compared with 0 min time point, ***P<0.001 at 5 min, **P<0.01 at 10-min, *P<0.05 at 15-min, n = 6 mice in each group. (D) Intraplantar injection of SB366791 (2.5-µg/10-µl) or amiloride (100-µg/10-µl), not DMSO (1%/10-µl), inhibited acid-induced thermal and mechanical hyperalgesia. *P<0.05, from 5 to 25-min time point, SB366791+pH 5.0 PBS group vs. DMSO+pH 5.0 PBS group. *P<0.05, from 15 to 25-min time point, amiloride+pH 5.0 PBS group vs. DMSO+pH 5.0 PBS group. Inset figures show that the calculated area under curve (AUC) (0–60-min) in PWL and PWT tests were significantly increased in SB366791+pH 5.0 PBS group and amiloride (100-µg/10-µl)+pH 5.0 PBS group. **P<0.01 compared with DMSO+pH 5.0 PBS group, n = 8 mice in each group. (E) Representative immunohistochemical staining and quantitative data of Fos in the spinal cord in mice. Intraplantar injection of SB366791 (2.5-µg/10-µl)+pH 5.0 PBS (10-µl), not amiloride(100-µg/10-µl)+pH 5.0 PBS and DMSO (1%/10-µl)+pH 5.0 PBS (10-µl) group decreased spinal Fos protein expression. Quantitative data indicats the number of Fos positive neurons in the spinal cord in each group. **P<0.01 compared with DMSO+pH 5.0 PBS group, n = 6 mice in each group. (F) The representative bands (top) for the expression of p-ERK after injection of SB366791 (2.5-µg/10-µl)+pH 5.0 PBS (10-µl), amiloride (100-µg/10-µl)+pH 5.0 PBS, or DMSO (1%/10-µl)+pH 5.0 PBS (10-µl) group and the quantitative data (bottom) for the expression of p-ERK. The fold change for the density of p-ERK is normalized to total-ERK for each sample. The fold change for the density of p-ERK levels in DMSO+pH 5.0 PBS group was set at 1 for quantifications. Compared with DMSO+pH 5.0 PBS group, *P<0.05, n = 6 mice in each group.

Previous studies have shown that H^+^ (low pH) produced hyperalgesia in animals and humans [8], [12]. In agreement with these reports, our results also show that intraplantar injection of pH 5.0 PBS, but not pH 7.4 PBS, induced thermal and mechanical hyperalgesia in mice which could last about 20-min and return to baseline level at 30-min after intraplantar injection of pH 5.0 PBS (Fig. 1A). Spinal neuronal sensitization was involved in the development and maintenance of hyperalgesia. Fos protein, the product of the c-fos immediate early gene, has been used as a maker for neuronal activation in the central nervous system [20], [21]. There is a positive correlation between the quantity of Fos protein expression and spinal neuronal activation induced by nociceptive stimuli. Recent data suggested the expression of spinal p-ERK could act as a better marker for central sensitization in pain studies [22]. To further clarify the algesic effect of intraplantar injection of pH 5.0 PBS, we investigated the change of spinal Fos protein at 2-h and p-ERK expression at 0–30 min after intraplantar injection of pH 5.0 PBS. We found that intraplantar injection of pH 5.0 PBS, and not pH 7.4 PBS, induced a remarkable increase of spinal Fos protein and p-ERK expression. The expression of Fos protein mainly distributed in I∼V lamina of the spinal cord (Fig. 1B). The increased expression of spinal p-ERK lasted about 15-min and peaked at the 5-min time point which was consistent with the pain behavior induced by pH 5.0 PBS (Fig. 1C). Many studies have shown that TRPV1 and ASIC participate in nociceptive information processing at the spinal cord level. Therefore, we asked whether TRPV1 or ASIC were involved in acid-induced hyperalgesia, the increased expression of spinal Fos, and p-ERK. To address this question, SB366791 (2.5-ug/10-ul), a TRPV1 antagonist, or amiloride (100-ug/10-ul), a non-selective ASIC antagonist, was injected 30-min before injection of pH 5.0 PBS. The results show that SB366791 could completely abolished pH 5.0 PBS-induced thermal and mechanical hyperalgesia and the increase of spinal Fos protein and p-ERK expression (Fig. 1D, E, F). Injection of amiloride did not produce analgesic effects at the 5-min and 10-min time points, however, analgesia appeared 15-min after injection of pH 5.0 PBS. Injection of amiloride did not inhibit the spinal Fos protein and p-ERK expression (Fig. 1D, E, F). This result was in agreement with some previous reports. Leffler et al reported that the main acid-sensor unmyelinated nociceptor in mice is TRPV1 [23]. Amiloride was less effective in reducing severe acidification (pH 5.0) -evoked pain [24]. These results further confirmed that acidic PBS induced TRPV1-mediated hyperalgesia and spinal neuron sensitization.

### Acidic QX-314 prevents acid-induced thermal hyperalgesia, spinal neuron sensitization, and generation of action potentials

**Figure 2:**
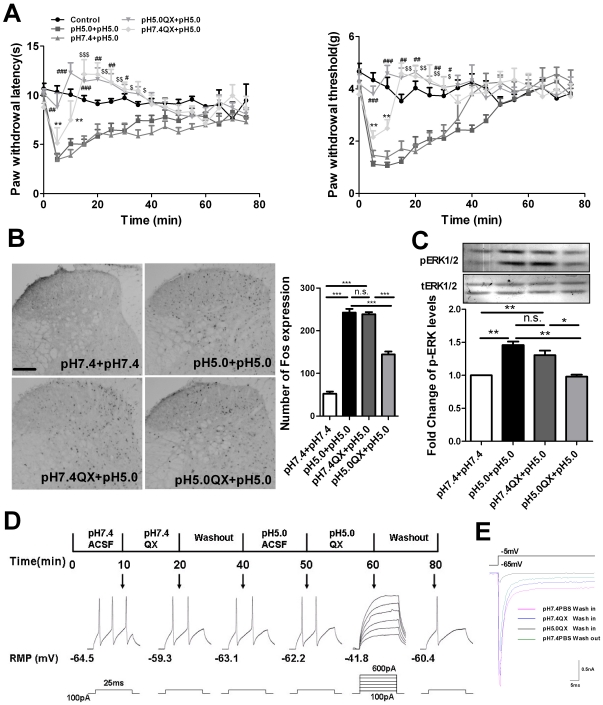
Acidic QX-314 inhibited acid-induced behavioral hyperalgesia and spinal neuronal sensitization. (A) Time course of thermal and mechanical hyperalgesia in control, pH 5.0 PBS+pH 5.0 PBS group, pH 5.0 QX-314+pH 5.0 PBS group, pH 7.4 PBS+pH 5.0 PBS group and pH 7.4 QX-314+pH 5.0 PBS group. The interval between the two injections was 15-min. **P<0.01 at 5-min and 10-min compared with control group, ^###^ *P*<0.001, ^##^ *P*<0.01, ^#^ *P*<0.05 at 5-min to 25-min point, ^$$^ *P*<0.01, ^$^ *P*<0.05 at 15-min to 30-min compared with pH 5.0 PBS+pH 5.0 PBS group or pH 7.4 PBS+pH 5.0 PBS, n = 8 mice in each group. (B) Representative immunohistochemical staining and quantitative data of Fos in the spinal cord in mice. Intraplantar pre-injection of pH 5.0 QX-314, but not pH 7.4 QX-314 attenuated the expression of spinal Fos protein induced by acid injection in mice. ***P<0.001, pH 7.4 PBS+pH 7.4 PBS group vs. pH 5.0 PBS+pH 5.0 PBS group, pH 7.4 QX-314+pH 5.0 PBS group vs. pH 5.0 QX-314+pH 5.0 PBS group, pH 5.0 QX-314+pH 5.0 PBS group vs. pH 5.0 PBS+pH 5.0 PBS group, n = 6 mice in each group. Scale bar = 100-µm. (C) The representative western blot bands (top) and the quantitative data (bottom) for the expression of p-ERK in the mouse spinal cord. The fold change for the density of p-ERK bands is calculated after normalization with t-ERK. p-ERK levels in pH 7.4 PBS+pH 7.4 PBS group were set at 1 for quantifications. **P<0.01, pH 7.4 PBS+pH 7.4 PBS group vs. pH 5.0 PBS+pH 5.0 PBS group, pH 5.0 QX-314+pH 5.0 PBS group vs. pH 5.0 PBS+pH 5.0 PBS, *P<0.05, pH 7.4 QX-314+pH 5.0 PBS group vs. pH 5.0 QX-314+pH 5.0 PBS group, n = 6 mice in each group. (D) Application of pH 5.0 QX-314 (5 -mM), but not pH 7.4 QX-314, blocked production of action potentials in primary DRG neurons. The first-forth and sixth panels: a depolarizing current step (100-pA, 25-ms) applied to small DRG neurons evoked a nociceptor-like broad action potential when it was in the solutions of pH 7.4 ACSF, pH 7.4 ACSF+QX-314, washout, pH 5.0 ACSF and washout. The fifth panel: pH 5.0 ACSF+QX-314 applied together completely abolished action potential generation even with a larger current injection (600-pA). (E) pH 5.0 QX-314 (5-mM), but not pH 7.4 QX-314, blocked total sodium current in DRG neurons. Total sodium current was recorded in DRG neurons by applying a depolarization voltage pulse from the holding potential of −65 mV to −5 mV in the voltage-clamp mode.

QX-314 could be delivered into the nociceptors via the activated TRPV1 channels by capcasin or chemical membrane permeation enhancers in different models [3], [4], [5], [6], [8]. Now that acidic PBS could activate the peripheral TRPV1 channels, acidic QX-314 should produce similar effects as capcasin. To test this speculation, an acid-evoked pain model was induced by intraplantar injection of pH 5.0PBS and pH 5.0 or pH 7.4 QX-314 (2%) 15-min before administration of pH 5.0PBS. Present results indicated that pretreatment with acidic QX-314 significantly inhibited acid-induced thermal and mechanical hyperalgesia behavior. Furthermore, paw thermal latency in the pH 5.0 QX-314 group was higher than its baseline level. pH 7.4 QX-314 did not produce an analgesic effect at the 5-min and 10-min time points. Its analgesic effect shown in the 15-min to 30-min time points should result from activating TRPV1 channels by the subsequent injection of pH 5.0PBS (Fig. 2A). The data from Fos and p-ERK expression further confirmed the behavioral results. Pretreatment with pH 5.0, not pH 7.4, QX-314 also significantly inhibited acid-induced increase of spinal Fos and p-ERK expression (Fig. 2B,C).

Blockage of voltage-gated Na^+^ channels could prevent the generation and propagation of action potentials. Next, we examined the effect of pH 5.0 and pH 7.4 QX-314 on action potentials evoked by current injection in cultured dorsal root ganglia by current patch clamp. After getting a small sized dorsal root ganglion (DRG) cell in a voltage patch clamp, we switched the recording mode form voltage patch clamp to current patch clamp and recorded resting membrane potential. 100-pA current was injected to produce action potentials in pH 7.4 ACSF. Then, pH 7.4 QX-314 was washed in for 10-min. 100-pA current injection could still induce action potentials. Next, we washed out pH 7.4 QX-314 for 20-min and washed in pH 5.0 ACSF for 10-min. The number of action potentials was less, but it could be evoked when the cells were bathed in pH 5.0 ACSF. Finally, we washed in pH 5.0 QX-314 for 10-min and found that current injection, even 6 times more, could not evoke the generation of action potentials. The effect of pH 5.0 QX-314 could be washed out (Fig. 2D). Finally, to investigate the effect of pH 5.0 QX-314 on sodium current, total sodium current was recorded in the voltage-clamp mode in DRG neurons by applying a depolarizing voltage pulse from the holding potential of −65 mV to −5 mV in the presence of potassium and calcium channel blockers. After recording a sodium current in pH 7.4 PBS, pH 7.4 QX-314 was washed in for 5-min; sodium current was elicited by this depolarizing voltage pulse although its amplitude was reduced slightly. However, sodium current was almost completely blocked by the following pH 5.0 QX-314 wash. This effect could be washed out by pH 7.4 PBS (Fig. 2E). These results were in accordance with behavioral and immunohistochemical findings and demonstrated that QX-314 could enter into cells and block sodium channels by delivery in an acidic solution.

### TRPV1, not ASIC, mediates the analgesic effects of acidic QX-314

**Figure 3:**
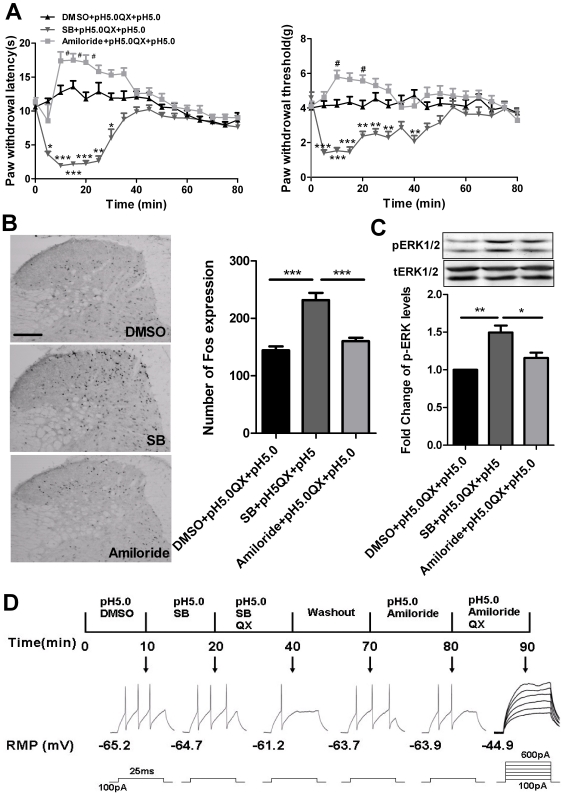
TRPV1 channels, but not ASIC, inhibited the analgesic property of acidic QX-314. (A) Time course of thermal and mechanical hyperalgesia in SB+pH 5.0 QX-314+pH 5.0 PBS group, DMSO+pH 5.0 QX-314+pH 5.0 PBS group and amiloride+pH 5.0 QX-314+pH 5.0 group. SB366791 (2.5-µg/10-µl) or amiloride (100-µg/10-µl) was given at 25-min before pH 5.0 QX-314 injection and at 40-min before pH 5.0 PBS injection, ***P<0.001, **P<0.01, *P<0.05 at 5-min to 25-min time point, and ^#^ *P*<0.05 at 10-min to 20-min time point compared with DMSO+pH 5.0 QX-314+pH 5.0 PBS group, n = 8 mice in each group. (B) Representative immunohistochemical staining of Fos in the spinal cord of mice in the DMSO+pH 5.0 QX-314+pH 5.0 PBS group, SB366791+pH 5.0 QX-314+pH 5.0 PBS group and amiloride+pH 5.0 QX-314+pH 5.0 PBS group. Quantitative data indicates the number of Fos positive neurons in the spinal cord in each group. ***P<0.001, DMSO+pH 5.0 QX-314+pH 5.0 PBS group vs. SB366791+pH 5.0 QX-314+pH 5.0 PBS group, SB366791+pH 5.0 QX-314+pH 5.0 PBS group vs. amiloride+pH 5.0 QX-314+pH 5.0 PBS group, n = 6 in each group. Scale bar = 100-µm. (C) p-ERK was examined at 10-min after pH 5.0 PBS injection, and the representative western blot bands (top) and the quantitative data (bottom) for the expression of p-ERK in the spinal cord of mice is shown. The fold change for the density of p-ERK bands is calculated after normalization with the DMSO+pH 5.0 QX-314+pH 5.0 PBS group. p-ERK levels in the DMSO+pH 5.0 QX-314+pH 5.0 PBS group was set at 1 for quantifications. **P<0. 01, DMSO+pH 5.0 QX-314+pH 5.0 PBS group vs. SB366791+pH 5.0 QX-314+pH 5.0 PBS group; *P<0. 05, SB366791+pH 5.0 QX-314+pH 5.0 PBS group vs. amiloride+pH 5.0 QX-314+pH 5.0 PBS, n = 6 mice in each group. (D) Application of SB366791 (10-µM), but not amiloride (100-µM), prevented the blockage effect of pH 5.0 QX-314 on production of action potentials in primary DRG neurons. The first-forth and sixth panels: a depolarizing current step (100-pA, 25-ms) applied to small DRG neurons evoked a nociceptor-like broad action potential when it was in the solutions of pH 5.0 ACSF+DMSO, pH 5.0 ACSF+SB366791, pH 5.0 ACSF+SB366791-QX-314, washout and pH 5.0 ACSF+amiloride. The sixth panel: pH 5.0 ACSF+amiloride-QX-314 applied together completely abolished action potential generation even with larger current injections (600-pA).

Both TRPV1 and ASIC are expressed in peripheral nociceptors and their cellular bodies DRG and could be activated by acid solution. Therefore, we want to know which one or if both channels were involved in the analgesic effect of acidic QX-314. To answer this question, SB366791 (2.5-ug/10-ul) or the ASIC antagonist amiloride (100-µg/10-ul) was injected at 25-min or 10-min before injection of pH 5.0 QX-314. We found that pretreatment with SB366791 completely prevented the analgesic effect of acidic QX-314 (Fig. 3A). However, pretreatment with amiloride could enhance the analgesic effect of acidic QX-314 (Fig. 3A). Furthermore, pretreatment with SB366791, not amiloride, could also prevent the inhibition of acid-induced Fos and p-ERK expression by acidic QX-314 (Fig. 3B,C). Next, we performed electrophysiological recordings to further confirm the above results. 100-pA current was injected to produce action potentials in cells treated with pH 5.0 DMSO. Then, pH 5.0 SB366791 was washed in for 10-min. 100-pA current injection still could induce action potentials. Next, we washed in pH 5.0 SB366791 QX-314 for 20-min and saw that action potentials still could be evoked. Finally, we washed out pH 5.0 SB366791 QX-314 for 30-min and washed in pH 5.0 amiloride QX-314 for 10-min and found that current injection, even 6 times more, could not evoke the generation of action potentials, suggesting TRPV1, not ASIC, channels mediated the analgesic effect of acidic QX-314 (Fig. 3D).

### Acidic QX-314 produces analgesic effect in a noradrenaline-induced pain model

**Figure 4:**
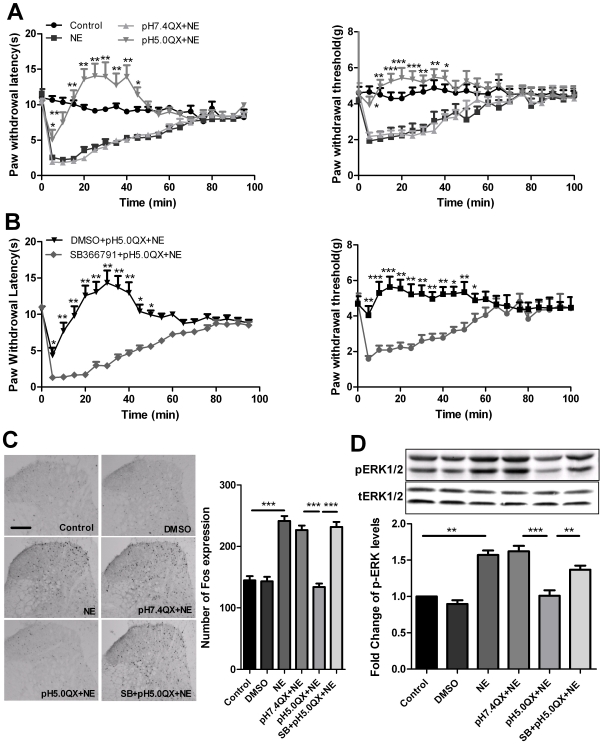
Acidic QX-314 inhibited NE-induced behavioral hyperalgesia and spinal neuronal sensitization. (A) Pre-treatment with pH 5.0 QX-314 (2.5-µg/10-µl) inhibited thermal and mechanical hyperalgesia induced by intraplantar injection of NE (0.5%, 10-µl). **P<0.01 or *P<0.05 at 5-min to 40-min time point compared with NE or pH 7.4 QX-314+NE group, n = 8 mice in each group. (B) Pre-injection of SB366791 prevented the analgesic effect of pH 5.0 QX-314 in NE-induced thermal hyperalgesia in mice. ***P<0.001, **P<0.01 or *P<0.05 at 5-min to 55-min time point compared with SB366791+pH 5.0 QX-314+NE, n = 8 mice in each group. (C) Intraplantar injection of pH 5.0 QX-314, but not pH 7.4 QX-314, attenuated the expression of spinal Fos protein induced by NE in mice, which could be abolished by pre-injection of SB366791. Representative immunohistochemical staining of Fos in the spinal cord of mice in the control group, DMSO group, NE group, pH 7.4 QX-314+NE group, pH 5.0 QX-314+NE group and SB366791+pH 5.0 QX-314+NE group. Quantitative data indicates the number of Fos positive neurons in the spinal cords of mice in each group. ***P<0.001, NE group vs. control group or DMSO group; pH 7.4 QX-314+NE group vs. pH 5.0 QX-314+NE group, pH 5.0 QX-314+NE group vs. SB366791+pH 5.0 QX-314+NE group, n = 6 mice in each group. Scale bar = 100-µm. (D) p-ERK was examined at 10-min after pH 5.0 PBS injection. The representative western blot bands (top) and the quantitative data (bottom) for the expression of p-ERK in the spinal cord of mice are shown. The fold change for the density of p-ERK bands is calculated after normalization with control. p-ERK levels in control group were set at 1 for quantifications. **P<0.01, NE group vs. control group or DMSO group, pH 5.0 QX-314+NE group vs. SB366791+pH 5.0 QX-314+NE group; ***P<0.001, pH 7.4 QX-314+NE group vs. pH 5.0 QX-314+NE group, n = 6 in each group.

Previous studies have shown that injection of noradrenaline (NE) can cause pain in human and animals [25], [26]. In the present experiment, we used this pain model to further confirm the analgesic effect of acidic QX-314. In agreement with previous reports, intraplantar injection of NE (0.5%, 10-µl) produced a significant thermal and mechanical hyperalgesic behavior in mice which could last at least 60-min (Fig. 4A). This injection also increased spinal Fos and p-ERK expression (Fig. 4C, D). Pretreatment with pH 5.0, but not pH 7.4, QX-314 at 15-min before injection of NE prevented NE-induced pain behavior and the increase of spinal Fos and p-ERK expression. Above effects could be abolished by pre-injection of TRPV1 inhibitor SB366791 (Fig. 4B, C, D). These results further demonstrated that acidic QX-314 could produce the analgesic effect mediated by TRPV1 channels.

### Sciatic nerve blockage with acidic QX-314 produces sensory-specific analgesic effects in naïve and chronic neuropathic pain in mice

**Figure 5:**
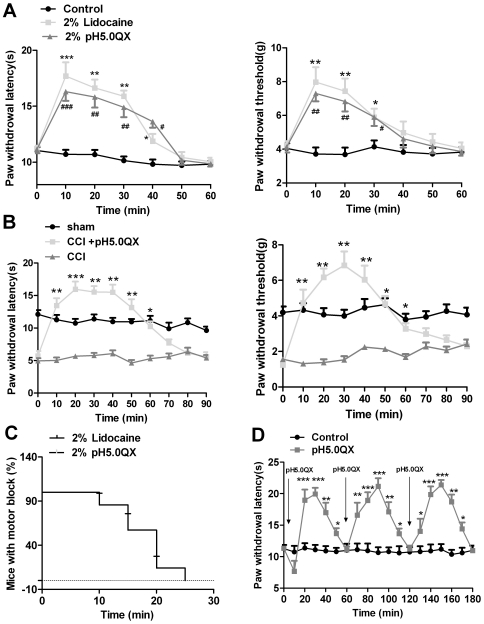
Sciatic nerve blockage with acidic QX-314 produced sensory-specific analgesic effect in naïve and chronic neuropathic pain mice. (A) Time course of thermal and mechanical hyperalgesia after injection of lidocaine (2%, 20 -µl) and pH 5.0 QX-314 (2%, 20 -µl) in the sciatic nerve blockage model in naïve mice. ***P<0.001, **P<0.01, *P<0.05 or ##*P*<0.01, #*P*<0.05 at 10-min to 40-min time point compared with control group, n = 8 mice in each group. (B) Time course of thermal and mechanical hyperalgesia after injection of pH 5.0 QX-314 (2%, 20-µl) in the sciatic nerve blockage model in CCI mice. ***P<0.001, **P<0.01, *P<0.05 in the 10-min to 60-min time point compared with CCI group, n = 8 mice in each group. (C) Injection of lidocaine, but not pH 5.0 QX-314 into the sciatic nerve produced motor blockage. The offset time was defined as the time point at which the mouse regained its ability to use the treated hind limb to hang on to the inverted mesh. Mice were tested 10-min after injection until recovery. Survival fractions were calculated using the product limit (Kaplan-Meier) method. (D) Time course of thermal hyperalgesia after repeated injection of pH 5.0 QX-314, ***P<0.001, **P<0.01, *P<0.05 at the 20-min to 170-min time point compared with control group. n = 6 mice in each group.

TRPV1 channels are not expressed in neurons of motor nerves. Therefore, QX-314 entry into cells mediated by capsaicin-activated TRPV1 channels only blocks sensory nerves and does not affect motor nerve function. Knowing that the analgesic effect of acidic QX-314 is mediated by TRPV1 channels, we predict that it should only block sensory nerves and have no effect on motor nerves. In the present study, we found that injection of acidic QX-314 (2%, 20-µl) into the popliteal space produced a significant sensory blockade without any impairment on movement. However, mice given a lidocaine injection experienced a 15–25-min paralysis. Both of the agents induced a similar sensory blockage for about 30-min and returned to the baseline level at 40-min after injection (Fig. 5A,B,E). Next, we wanted to know whether injection of acidic QX-314 also produced analgesic effect in chronic pain status. To address this question, a neuropathic pain model induced by chronic constrictive injury (CCI) was performed and 2% acidic QX-314 was injected into the popliteal space at 5 days after CCI, and then thermal hyperalgesia and mechanical allodynia were measured at different time points after injection of acidic QX-314. We found that acidic QX-314 produced a significant analgesic effect without any impairment of motor nerve function (Fig. 5C,D). In some clinical cases, LA needs to be employed repeatedly. To test whether the repeated injection of acidic QX-314 could produce a similar effect for each time, we intraplantarly injected pH 5.0 QX-314 three times at intervals of 60-min. The present results showed each injection had a similar time-course and intensity of sensory blockage (Fig. 5E).

## Discussion

The present study showed the following findings (1) acidic QX-314 prevented acid or NE-induced thermal and mechanical hyperalgesia and the increase of spinal Fos and p-ERK expression, which could be abolished by TRPV1 antagonist SB366791 and not by the ASIC antagonist amiloride. (2) pH 5.0, not pH 7.4, QX-314 blocked sodium currents and abolished the current injection-evoked generation of action potentials in DRG neurons; the latter could be prevented by SB366791 and not by amiloride. (3) acidic QX-314 produced the analgesic effect without impairment of motor functions in mouse sciatic nerve blockage model in naïve and CCI mice. These findings indicated that acidic QX-314 selectively blocks sensory nerves mediated by a TRPV1-dependent mechanism.

LA have covered a long history since cocaine was first employed in clinics in the late 19^th^ Century, and increasingly more LA have appeared since then [27]. Almost of all LA produced analgesic effects by interrupting neuron excitation and conduction via blockage of voltage-gated sodium channels. Therefore, it was unavoidable to impair movement as well as block pain sensation. Recently, Woolf and colleagues reported that co-administration of capsaicin and QX-314 produces a long-lasting sensory-specific blockade [3]. Accumulated evidence has built the theory that QX-314 enters into the nociceptors via activated-TRPV1 channels or surfactants-induced penetration of the cell membrane and blocks the Na^+^ channels from the intracellular side [3], [4], [5], [6], [7]. What we know is that QX-314 acts as a local anesthetic only when it is delivered into the nociceptors. However, these methods of drug combination would induce some side effects and also were inconvenient to use. In the present study, we found that acidic QX-314 can produce selective analgesia similar to those combinations. The pH value of clinically-used LA such as lidocaine and bupivacaine is pH 3-5.5 in hydrochlorate or carbonate form. So, the pH value of the solution used in this study is acceptable within those limits.

There was a viewpoint that low pH solution injected into peripheral tissue was buffered quickly and was unsuitable as a medium for introducing QX-314 intracellularly [4]. In this study, we found that injection of pH 5.0 PBS resulted in marked thermal hyperalgesia (lasting for 15–20-min) and sensitization of spinal neurons manifesting as activation of spinal p-ERK and c-Fos, which could be prevented by pretreatment with a TRPV1 antagonist, indicating injection of acidic solution peripherally could activate TRPV1 channels. Based on the results of our behavioral test, spinal p-ERK, and Fos expression, we thought that intraplantar injection of 10-µl acidic solution had enough time to activate TRPV1 channels or other channels like ASIC before it was buffered, which usually is an immediate process, and TRPV1-mediated downstream activation (for example, ERK signaling pathway) would keep the TRPV1 channels open at later time points to save enough time for QX-314 entering intracellularly. Furthermore, we found that acidic QX-314 and not neutral pH QX-314 alleviated acid- and NE-induced thermal hyperalgesia, inhibited spinal p-ERK and Fos expression, and abolished production of action potentials. Next, we injected acidic QX-314 into the popliteal space and found that this injection induced a significant sensory blockage without any impairment of movement. These results provide strong evidence that acidic solution can be used as medium for introducing QX-314 into cells, which could produce a selective analgesic effect *in vivo*. The duration of analgesic effect of acidic QX-314 was shorter than what was induced by capsaicin-activated TRPV1 channels or by chemical membrane permeation enhancers [3], [28], [29]. However, the similar time-course and intensity of blockade produced by repeated injection could make up for the short-acting deficiency of QX-314. Actually, short-acting LA are also useful in some clinical cases. We also intrathecally injected acidic QX-314 and found that some of the mice that received this injection died after an immediate irritation (data not shown), which is consistent with a recent study reported by Schwarz et al [30]. We will investigate the reason and mechanisms of this phenomenon in following experiments.

The present study found that acidic QX-314 prevents acid- and NE-induced thermal and mechanical hyperalgesia, spinal neuron sensitization and production of action potentials, which could be prevented by SB366791, a TRPV1 antagonist, but not by amiloride, an antagonist of ASIC. Both TRPV1 and ASIC channels were activated by acid injection and involved in pain behavior. We didn't investigate which channel prominently contributed to the acid-induced pain. However, TRPV1 channels, not ASIC, mediated the analgesic property of acidic QX-314.

In conclusion, we have demonstrated that administration of acidic QX-314 can provide a sensory-selective nerve blockage in different models. These findings have important implications for exploring QX-314 as a novel LA and suggest that acidic QX-314 solution may have potential for clinical use.
